# Access to Cervical Cancer Screening for Women Living With HIV: A Record Linkage Study.

**DOI:** 10.23889/ijpds.v7i3.2032

**Published:** 2022-08-25

**Authors:** Mushashu Lumpa, Miquel Duran-Frigola, Albert Manasyan, Jane Matambo, Mulindi Mwanahamuntu, Samuel Bosomprah, Izukanji Sikazwe, Kevin Fahey

**Affiliations:** 1 University of Bern, Bern. Switzerland; 2 Swiss Tropical and Public Health Institute, Allschwil, Basel, Switzerland; 3 CIDRZ, Lusaka. Zambia; 4 Ersilia; 5 University of Alabama at Birmingham, AL, USA; 6 Centre for Infectious Disease Research in Zambia, Lusaka. Zambia; 7 Women and New Born Hospital, University Teaching Hospital, Lusaka. Zambia; 8 University of Basel, Basel, Switzerland

## Objectives

According to the Global Strategy to Accelerate the Elimination of Cervical Cancer launched by the World Health Organizations (WHO), 70% of all women should undergo cervical cancer (CC) screening by 2030. While integrated HIV care and CC screening services exist, lack of unified data and patient management systems hinder reaching the set WHO goals. We used record linkage methods to assess the number of women receiving HIV care and visual inspection with acetic acid (VIA) based CC screening at Kanyama First Level Hospital (FLH) in Lusaka, Zambia.

## Approach

We retrieved HIV patient records from SmartCare, Zambia’s National Electronic Health Records system, and the CC screening database for the years 2004 to 2018, and 2010 to 2018, respectively. We restricted this pilot study to women attending Kanyama FLH for HIV services, and its surrounding clinics for CC screening. We used the following linkage variables: first name, last name, facility, and year of birth. We used a machine learning based approach with Random Forest classifiers to identify records from the HIV and CC screening services belonging to the same person. We created synthetic patient record datasets to train and test the linkages. After the linkage, we anonymized the data and retrieved screening status from the CC screening database. We assessed the percentage of women in HIV care undergoing CC screening and the respective screening results. We calculated the time to CC screening with reference to the start of the HIV Antiretroviral Therapy treatment (ART).

## Results

We retrieved a total of 32,970 records from SmartCare and 176,736 records from CC screening databases. With the linkage we determined that of all women that ever accessed HIV treatment services at Kanyama FLH between 2004 and 2018, 2,999 (9%) received CC screening. Of those that were screened, 30% received the CC service at Kanyama FLH, while the rest from 13 surrounding facilities. 61% of women accessed CC services before starting ART. Of the women screened for CC, 371 had a positive VIA result (12%), 2,395 (80%) a negative and 233 (8%) had a missing or indeterminate result. When we restricted the analysis to women, who were eligible for screening (aged 25 to 59 years), the screening coverage was 9.6%.

## Conclusion

Our study demonstrates the significance of record linkage efforts in determining CC screening coverage among women living with HIV. According to these findings, CC screening coverage among patients receiving ART services between 2004 and 2018 was at 9%, showing the depth of effort required to meet WHO targets at the time. The linkage output was primarily influenced by the scarcity of available linkage variables and data missingness, resulting in potentially overestimating or underestimating the CC coverage. This is typical of data systems in low- and middle-income countries; however, this study demonstrates that record linkage is useful in bridging these widespread data gaps.

